# Microplastics increase cadmium absorption and impair nutrient uptake and growth in red amaranth (*Amaranthus tricolor* L.) in the presence of cadmium and biochar

**DOI:** 10.1186/s12870-024-05312-0

**Published:** 2024-06-26

**Authors:** Rana Roy, Akram Hossain, Shirin Sultana, Biplob Deb, Md. Moudud Ahmod, Tanwne Sarker

**Affiliations:** 1 Institute of Plant Nutrition and Soil Science, Christian-Albrechts-Universität zu Kiel, 24118 Kiel, Germany; 2 Department of Agroforestry and Environmental Science, Sylhet Agricultural University, Sylhet, 3100 Bangladesh; 3 Open School, Bangladesh Open University, Gazipur, 1705 Bangladesh; 4 Department of Agricultural Extension Education, Sylhet Agricultural University, Sylhet, 3100 Bangladesh; 5 Department of Crop Botany & Tea Production Technology, Sylhet Agricultural University, Sylhet, 3100 Bangladesh; 6 Department of Sociology and Rural Development, Khulna Agricultural University, Khulna, 9100 Bangladesh

**Keywords:** Polyethylene, Polyethylene terephthalate, Polystyrene, Cadmium, Biochar, Leafy vegetable, Combined pollution

## Abstract

Microplastic (MP) pollution in terrestrial ecosystems is gaining attention, but there is limited research on its effects on leafy vegetables when combined with heavy metals. This study examines the impact of three MP types—polyethylene (PE), polyethylene terephthalate (PET), and polystyrene (PS)—at concentrations of 0.02, 0.05, and 0.1% w/w, along with cadmium (Cd) and biochar (B), on germination, growth, nutrient absorption, and heavy metal uptake in red amaranth (*Amaranthus tricolor* L.). We found that different MP types and concentrations did not negatively affect germination parameters like germination rate, relative germination rate, germination vigor, relative germination vigor, and germination speed. However, they increased phytotoxicity and decreased stress tolerance compared to an untreated control (CK1). The presence of MPs, particularly the PS type, reduced phosphorus and potassium uptake while enhancing Cd uptake. For example, treatments PS_0.02_CdB, PS_0.05_CdB, and PS_0.1_CdB increased Cd content in *A. tricolor* seedlings by 158%, 126%, and 44%, respectively, compared to the treatment CdB (CK2). Additionally, MP contamination led to reduced plant height, leaf dry matter content, and fresh and dry weights, indicating adverse effects on plant growth. Moreover, the presence of MPs increased bioconcentration factors and translocation factors for Cd, suggesting that MPs might act as carriers for heavy metal absorption in plants. On the positive side, the addition of biochar improved several root parameters, including root length, volume, surface area, and the number of root tips in the presence of MPs, indicating potential benefits for plant growth. Our study shows that the combination of MPs and Cd reduces plant growth and increases the risk of heavy metal contamination in food crops. Further research is needed to understand how different MP types and concentrations affect various plant species, which will aid in developing targeted mitigation strategies and in exploring the mechanisms through which MPs impact plant growth and heavy metal uptake. Finally, investigating the potential of biochar application in conjunction with other amendments in mitigating these effects could be key to addressing MP and heavy metal contamination in agricultural systems.

## Introduction

Plastics, a ubiquitous type of material known for its malleability and wide range of applications, have become an essential component of modern life [1]. These synthetic and semi-synthetic materials, however, are at the root of a growing environmental crisis [2]. Geyer et al. have predicted that by 2050, approximately 12,000 million metric tons of plastic waste will accumulate in landfills and natural environments [3]. Plastic infiltration into soils occurs from various sources, including packaging materials, sewage treatment plants, disposable products, and agricultural mulching [4]. When plastics are exposed to environmental factors like heat and sunlight for long periods, they become brittle and fracture, producing smaller particles through physical, chemical, and biological processes [5]. Certain fungi have also evolved to break down specific plastic polymers and produce microplastics [2]. Microplastics (MPs), defined as particles smaller than 5 mm, are recognized as a growing pollutant worldwide [6, 7]. It has been shown that MPs affect soil bulk density, water-holding capacity, aggregates, porosity, structure, and soil organisms [8, 9]. The decline in seed germination viability, inhibition of plant growth, and even impairment of food quality have also been documented in earlier research as possible effects of MPs [10–12]. It is anticipated that the concentration of MPs will continue to rise in the coming years, making it critical to research the potential impact of various plastic polymers on plant species [7, 9, 13].

Like MPs, the presence of metals and metalloids is a typical occurrence in agricultural soils [14, 15]. Cadmium (Cd) contamination is particularly prevalent in Bangladesh, a country with an extremely high population density [16]. Since MPs and Cd are commonly found together, there is a possibility that they may interact to influence the metal’s bioavailability and toxicity in agricultural ecosystems [17]. Specifically, MPs have the ability to absorb metal pollutants, making them a potential route for the entry of these metals into living organisms [1]. However, studies have shown that organic additions, such as biochar, can help plants thrive, increase soil fertility, and even immobilize heavy metals [13, 18].

Biochar is a promising remediation agent for various pollutants, including organic compounds, heavy metals, and toxic substances [19]. Its unique physicochemical properties, including large surface area, increased porosity, and functional groups, make it an environmentally friendly solution for the removal of various toxins [20]. Biochar has been shown to have better adsorptive properties than typical carbonaceous adsorbents when it comes to removing MPs from soil [9]. The adsorption capacity and structural properties of biochar are crucial factors for the effective removal of MPs in this process. The effectiveness of biochar in mitigating the negative effects of MPs in both aqueous and terrestrial environments is also reported in several studies [13, 21, 22]. Tursi et al. [23] have shown that biochar can be a cost-effective solution for MP removal. Applying biochar to soils contaminated with MPs not only helps to remove the contaminants, but it can also enhance soil quality and promote plant development [13]. MPs serve as carriers for metal absorption in plants, while biochar concurrently decreases the bioavailability of toxic metals in soils [24]. Consequently, questions emerge about what happens when MPs, Cd, and biochar coexist and how this affects plant growth.

Given these considerations, the effects of varying concentrations of three distinct types of microplastics—namely polyethylene (PE), polyethylene terephthalate (PET), and polystyrene (PS)—in the presence of cadmium (Cd) and biochar were assessed for their impact on the growth of red amaranth (*Amaranthus tricolor* L.) seedlings. The PE, PET, and PS are three typical MP types commonly detected in agricultural soil, which have consequences on the seed germination and seedling growth of several plant species [25–27]. Therefore, we selected these three MP types for our study. Furthermore, we specifically selected *A. tricolor* as our focus plant species due to its widespread consumption as a vegetable in Bangladesh. The *A. tricolor* is a pseudo-cereal crop with both food and health benefits [28, 29]. As a source of phenols, minerals, plant pigments, vitamins, and flavonoids, *A. tricolor* can provide bioactive compounds, antioxidants, and essential nutrients [30–32]. The plant also produces betacyanin, a water-soluble nitrogenous pigment, which can be used as a raw material for natural pigment production [33]. The *A. tricolor* is an emerging crop with great economic value. The purpose of this study was to determine how *A. tricolor* was affected in terms of germination, growth characteristics, nutrient absorption, and heavy metal uptake by three distinct types and concentrations of MPs in the presence of Cd and biochar. We hypothesized that exposure to different types and increasing levels of MP concentrations in the presence of Cd and biochar would (a) reduce germination, growth characteristics, chlorophyll content, and nutrient absorption, and (b) increase Cd uptake, producing synergistic ecotoxicity effects in *A. tricolor*.

## Materials and methods

### Pot experiment

Pot experiment was carried out in a without temperature controlling plastic shed at the Sylhet Agricultural University, Bangladesh (24° 54′ 33.12″ N, 91° 54′ 7.2″ E). Soil was collected from the local area at a depth of 0–15 cm. Soils were stack in net house and air dried. Prior to potting, soil was ground and mixed well, and properties were determined. Properties of experimental soil and biochar before starting the experiments were presented in Table 1.

**Table 1:** Chemical properties of soil samples and biochar before starting the experiment

Treatment	pH (1:2.5, soil: water)	OM (%)	OC (%)	Total-N (%)	Available P	Exchangeable K	Total Pb (mg kg^− 1^)	Total Cd (mg kg^− 1^)
Soil	5.2	2.58	1.5	0.129	23.05 mg kg^− 1^	0.28 (meq 100 g^− 1^ soil)	4.25	0.047
Biochar	11.01	-	7.28	0.626	0.81%	0.98%	5.66	0.029

Exactly 500 g of air-dry soil was taken into square-shaped polyethylene terephthalate (PET) plastic pots (9.5 × 9.5 cm at top, 8.0 × 8.0 cm at bottom, 8.0-cm height) and different types and concentrations of MPs were added with the soil according to treatment details. We added cadmium chloride monohydrate (CdCl_2_·H_2_O) to achieve a Cd concentration of 5.0 mg kg^− 1^ of soil. Afterward, we applied biochar, produced from wood pyrolyzed at 400 °C, at a rate of 5.0 tons ha^− 1^. After that pots were watered well and left for 2 weeks for incubation.

Three distinct MP types like PE, PET, and PS were chosen for our investigation and acquired from Zhonglian Plastic Raw Material Company in Guangdong, China. The PE, PET, and PS MPs were sieved using 150 μm sieves. To reduce microbiological contamination, the PE, PET, and PS MPs were sterilized for 20 min on an ultraviolet clean bench [34]. Based on the environmentally realistic MP concentrations described in the literature [35–37], three concentrations of each MP type (e.g., 0.02%, 0.05%, and 0.1%, w/w MP/soil) were selected. In total 11 treatments were developed including two control treatments [CK2 (no MPs, only Cd and biochar), CK1 (no MPs, Cd and biochar)]. Each treatment was repeated three times.

Red amaranth var. BARI Lalshak-1 (*A. tricolor* L.) was selected for this study, with seeds sourced from the Bangladesh Agricultural Development Corporation in Mymensingh, Bangladesh. Twenty seeds were sown on October 14, 2022, and after two weeks of germination, only five healthy seedlings were kept per pot. Various fertilizers, including urea, triple superphosphate, and muriate of potash, were applied during soil preparation according to the Bangladesh Fertilizer Recommendation Guide [38]. Seedlings were harvested 40 days after sowing, on November 24, 2022. Throughout the growing season, we ensured that the seedlings received adequate water by watering them at regular intervals. The pots were examined regularly, and extra water was provided as needed to keep the soil moist without overwatering. This guaranteed that the seedlings remained healthy during the experiment.

### Measurement of growth parameters

The germination parameters measured in this experiment using the following formulae [39–41]:

1 $$\eqalign{& {\rm{Germination}}\,{\rm{rate}}\left( {{\rm{GR}}} \right) = \cr & {{Ng\,at\,day\,7} \over {Nt}} \times 100 \cr}$$

2 $$\eqalign{& {\rm{Germination}}\,{\rm{vigor}}\left( {{\rm{GV}}} \right){\rm{ = }} \cr & {{Ng\,at\,day\,3} \over {Nt}} \times 100 \cr}$$

3 $$\eqalign{& {\rm{Germination}}\,{\rm{speed}}\left( {{\rm{GS}}} \right){\rm{ = }} \cr & {{Ng\,at\,day\,3} \over {Ng\,at\,day\,7}} \times 100 \cr}$$

4 $$\eqalign{& {\rm{Vigour}}\,{\rm{index}}\left( {{\rm{VI}}} \right){\rm{ = }} \cr & ({\rm{GR}} \times {\rm{seedling}}\,{\rm{length}}) \cr}$$

5 $$\eqalign{& {\rm{Phytotoxicity }}\left( {{\rm{PHY}}} \right) = \cr & {{SLCK - SLT} \over {SLCK}} \times 100 \cr}$$

6 $$\eqalign{& {\rm{Stress}}\,{\rm{tolerance}}\,{\rm{index}}\,\left( {{\rm{STI}}} \right){\rm{ = }} \cr & {{SLT} \over {SLCK}} \times 100 \cr}$$

Where Ng is the number of germinated seeds, N_t_ was the total number of tested seeds. *SLCK* represents shoot length of control and *SLT* represents shoot length of specific treatment at 7^th^ day.

Various growth parameters were measured, including plant height (PH, cm), leaf dry matter content (LDMC, g g^− 1^), plant fresh weight (FW, g plant^− 1^), plant dry weight (DW, g plant^− 1^), root: shoot ratio, and relative water content (RWC, %) [42, 43]. The SPAD (Soil Plant Analysis Development) method was used to determine the chlorophyll content of *A. tricolor*. The evaluation was performed using a portable Minolta chlorophyll meter (SPAD-502, Osaka 590–8551, Japan). The SPAD values of chlorophyll content represent the mean for each treatment and were calculated using random samples of 3–5 fresh leaves from each plant.

To measure nutrient elements plant (leaf and stem) powder of 0.25 g was mixed with 0.5% nitric acid in a 50-ml beaker. After 24 h in an 80 °C water bath, the solution was filtered and diluted with deionized water. Mineral and nutrient elements were analyzed using an Inductively Coupled Plasma Spectrometer (ICPS-8100, Shimadzu Co. Ltd.). Soil and amaranth nitrogen content was determined with a Gas Chromatograph (Soil GS-8 A, Shimadzu Co. Ltd., NC-220 F Juka analysis center) and Sumigraph (NC-90 A, Shimadzu Co. Ltd.). Cd content in soil and *A. tricolor* (leaf, stem, and root) was determined using ICP-MS (Japanese-made Agilent 7900). The Bioconcentration factor (BCF) and translocation factor (TF) of Cd were calculated according to Sikdar et al. [44]. The BCF leaf, BCF stem, BCF root, and TF of Cd were calculated as follows: Cd_leaf_/Cd_soil_, Cd_stem_/Cd_soil_, Cd_root_/Cd_soil_, and Cd_(leaf+stem)_/Cd_root_, respectively.

The root system scanning operation was carried out with the help of a specialist root scanner (STD4800 Scanner) and WinRHIZO Pro software (Regent Instruments). Upon removing the plant from its pot, the roots were separated using sharp scissors, and subsequently, they were washed and positioned in water on a waterproof tray. The positioning of the roots was arranged in a manner that prevented any overlapping of lateral roots and ensured a randomized distribution. The root parameters were analyzed using the ‘Analysis’ mode. In the WinRHIZO system, the following parameters were produced: total root length (cm), root volume (cm^3^), root surface area (cm^2^), root diameter (mm), and the number of tips [45].

### Statistical analysis

Statistical analysis was performed using a statistical package, Statistix 8.0 (Analytical Software 2105 Miller Landing Rd. Tallahassee, FL 32,312). The presented data in Figures and Tables represent means ± standard errors (SEs) of three repetitions for each treatment. A one-way Analysis of Variance (ANOVA) was used to compare treatments, followed by Duncan’s multiple range tests at a 5% level of significance.

## Results

### Germination index

**Fig. 1:**
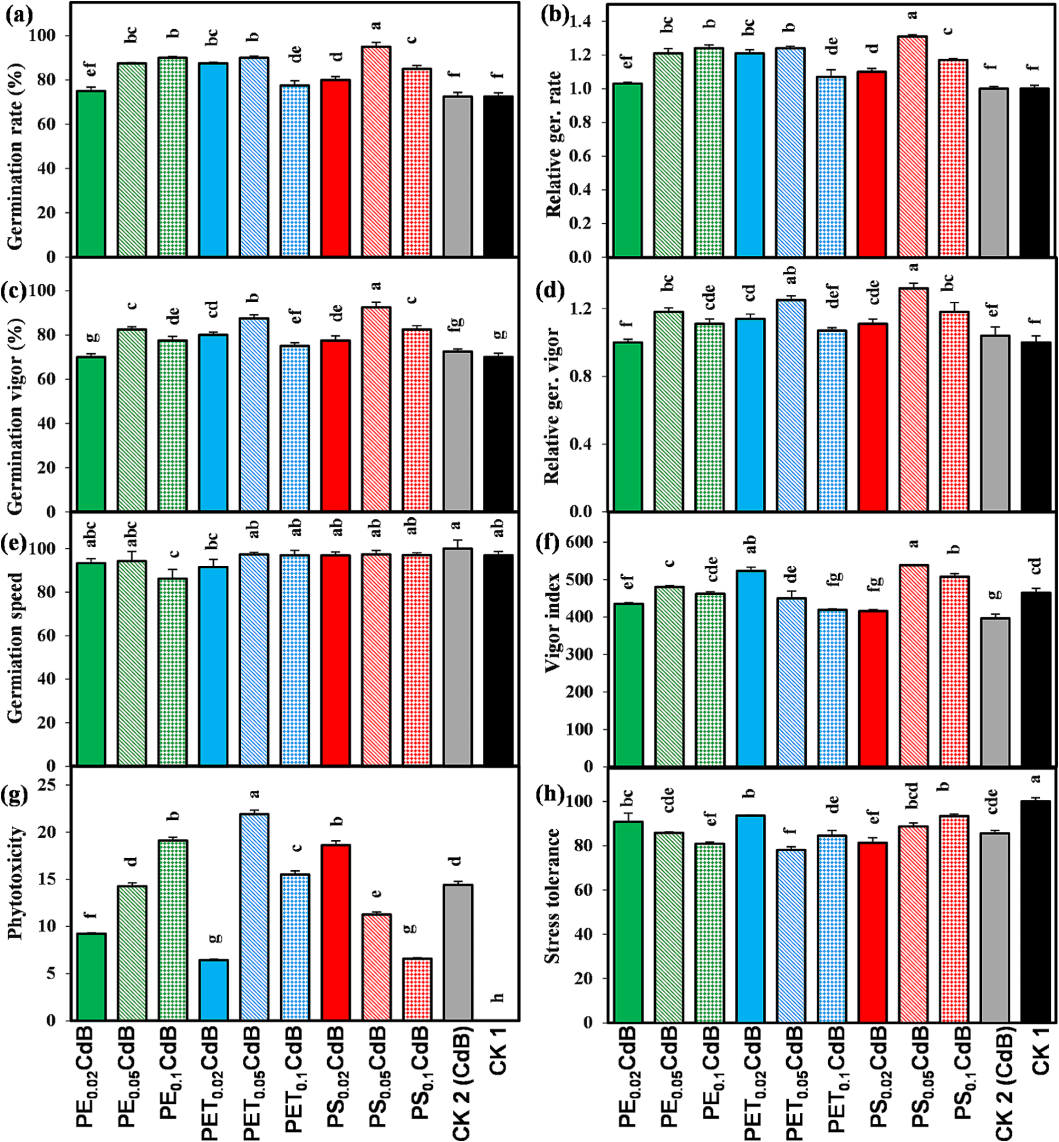
Effects of polyethylene (PE), polyethylene terephthalate (PET), and polystyrene (PS) on (**a**) germination rate, (**b**) relative germination rate, (**c**) germination vigor, (**d**) relative germination vigor, (**e**) germination speed, (**f**) vigor index, (**g**) phytotoxicity, and (**h**) stress tolerance of *A. tricolor* in presences of Cd and biochar (**B**). Different letters over the bars (**a, b, c, d**, etc.) indicate significant differences using a one-way ANOVA followed by Duncan’s multiple range test (*p* < 0.05) (*n* = 3)

The germination of *A. tricolor* was notably influenced by the types and concentrations of microplastics (MPs). In comparison to the control groups (CK1 and CK2), all treatments, with the exception of PE_0.02_CdB, exhibited a significant (*p* < 0.05) increase in germination rate, relative germination rate, germination vigor, and relative germination vigor (Fig. 1a-d). Notably, the germination speed in PE_0.1_CdB was significantly lower than that in CK1 and CK2, while other treatments showed no evident effects on germination speed (Fig. 1e). Treatments involving PET_0.02_CdB, PS_0.05_CdB, and PS_0.1_CdB resulted in a significant (*p* < 0.05) increase in vigor index compared to the controls (Fig. 1f). Moreover, when compared to the untreated control (CK1), *A. tricolor* exhibited a significant (*p* < 0.05) increase in phytotoxicity and a decrease in stress tolerance across all treatments (Fig. 1g-h). Overall, most MPs-containing treatments significantly enhance germination rate, relative germination rate, germination vigor, and relative germination vigor in *A. tricolor*, while increasing phytotoxicity and lowering stress tolerance as compared to the untreated control (CK1) (Fig. 1a-h).

### Morphological parameters, SPAD value and leaf water content

**Fig. 2:**
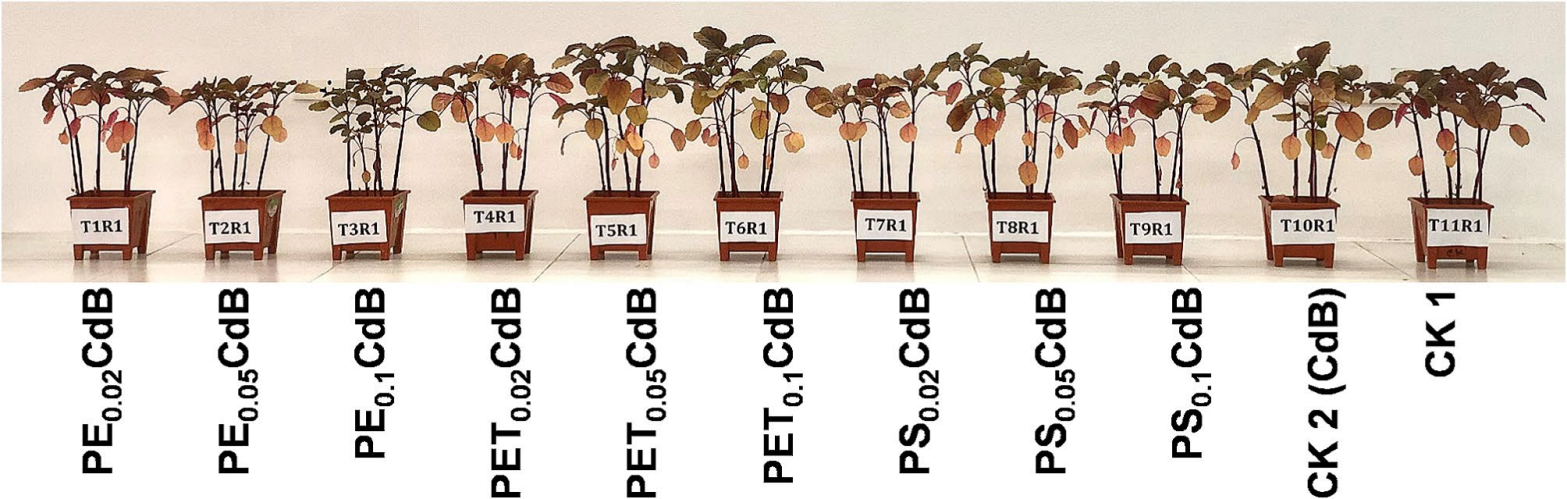
Effects of polyethylene (PE), polyethylene terephthalate (PET), and polystyrene (PS) on phenotypic expression of *A. tricolor* in presences of Cd and biochar (**B**)

Plant height showed a significant decrease (*p* < 0.05) in most treatments compared to the control group (CK1), except for PET_0.05_CdB, PET_0.1_CdB, and PS_0.05_CdB (Fig. 2; Table 2). Similarly, leaf dry matter content (LDMC) was significantly lower (*p* < 0.05) across all treatment groups relative to CK1 (Table 2). Furthermore, except for PET_0.05_CdB, both the fresh (FW) and dry weights (DW) of *A. tricolor* were significantly reduced (*p* < 0.05) across all treatments when compared to the control groups CK1 and CK2 (Table 2). The study found significant decreases in plant height, leaf dry matter content, and fresh and dry weights of *A. tricolor* in most treatments compared to the control groups CK1 and CK2 (Table 2).

**Table 2:** Effects of polyethylene (PE), polyethylene terephthalate (PET), and polystyrene (PS) on plant height (PH), leaf dry matter content (LDMC), fresh weight (FW), dry weight (DW), root: shoot (R: S) ratio, SPAD value and relative water content (RWC) of *A. Tricolor* in presences of Cd and biochar. Different letters (a, b, c, d, etc.) indicate significant differences using a one-way ANOVA followed by Duncan’s multiple range test (*p* < 0.05) (*n* = 3)

Treatments	PH	LDMC	FW	DW	*R*: S	SPAD	RWC
	cm	g g^− 1^	g plant^− 1^	g plant^− 1^			%
PE_0.02_CdB	23.5 ± 0.14^b^	0.15 ± 0.01^de^	3.5 ± 0.03^bc^	0.67 ± 0.03^cd^	0.29 ± 0.02^ab^	22.13 ± 0.32^f^	97.03 ± 1.71^ab^
PE_0.05_CdB	20.74 ± 0.11^de^	0.18 ± 0.01^b^	3.83 ± 0.04^b^	0.69 ± 0.07^bcd^	0.27 ± 0.02^ab^	22.73 ± 0.4^ef^	92.5 ± 2.26^b^
PE_0.1_CdB	20.12 ± 0.45^e^	0.17 ± 0.02^bcd^	2.98 ± 0.02^c^	0.56 ± 0.09^d^	0.31 ± 0.01^a^	22.7 ± 0.55^ef^	95.45 ± 2.97^ab^
PET_0.02_CdB	23.56 ± 0.57^b^	0.17 ± 0.01^bc^	3.8 ± 0.03^b^	0.72 ± 0.09^bc^	0.26 ± 0.01^ab^	25.33 ± 0.64^bc^	93.97 ± 2.68^ab^
PET_0.05_CdB	25.68 ± 0.43^a^	0.18 ± 0.03^b^	3.91 ± 0.28^b^	0.82 ± 0.06^ab^	0.23 ± 0.01^b^	26.6 ± 0.45^ab^	98.1 ± 2.65^ab^
PET_0.1_CdB	25.88 ± 0.42^a^	0.16 ± 0.01^cd^	3.64 ± 0.07^b^	0.72 ± 0.03^bc^	0.26 ± 0.02^ab^	27.73 ± 0.75^a^	98.73 ± 0.84^a^
PS_0.02_CdB	21.28 ± 0.36^cd^	0.18 ± 0.02^b^	3.59 ± 0.06^bc^	0.69 ± 0.04^bcd^	0.28 ± 0.02^ab^	23.53 ± 0.2^ef^	93.85 ± 2.25^ab^
PS_0.05_CdB	24.76 ± 0.49^a^	0.16 ± 0.01^cd^	3.57 ± 0.28^bc^	0.62 ± 0.03^cd^	0.27 ± 0.02^ab^	26.17 ± 0.63^bc^	93.76 ± 0.75^ab^
PS_0.1_CdB	22.31 ± 0.4^c^	0.16 ± 0.01^cd^	3.53 ± 0.03^bc^	0.71 ± 0.02^bcd^	0.29 ± 0.04^ab^	19.27 ± 0.15^g^	93.78 ± 1.75^ab^
CK 2 (CdB)	25.68 ± 0.34^a^	0.14 ± 0.02^e^	4.93 ± 0.24^a^	0.97 ± 0.02^a^	0.23 ± 0.02^b^	23.73 ± 0.44^de^	98.01 ± 1.39^ab^
CK 1	24.81 ± 0.21^a^	0.2 ± 0.01^a^	4.66 ± 0.52^a^	0.93 ± 0.01^a^	0.24 ± 0.03^b^	25 ± 0.57^cd^	95.23 ± 0.88^ab^

* Treatment nomenclature indicates the types and amount of microplastic application, designated as a subscript [e.g., PE_0.02_CdB = Polyethylene @ 0.02% in presence of Cd (Cadmium) and B (Biochar)]

The root: shoot ratio increased significantly under PE_0.1_CdB treatment, while other treatments did not show any significant effects. (Table 2). All concentrations of PET and PS_0.05_CdB significantly enhanced SPAD value, whereas other treatments decreased it as compared to CK1 (Table 2). Different MPs containing treatments did not significantly affect the RWC of *A. tricolor* compared to the controls (Table 2).

### Nutrient and heavy metal content

**Fig. 3:**
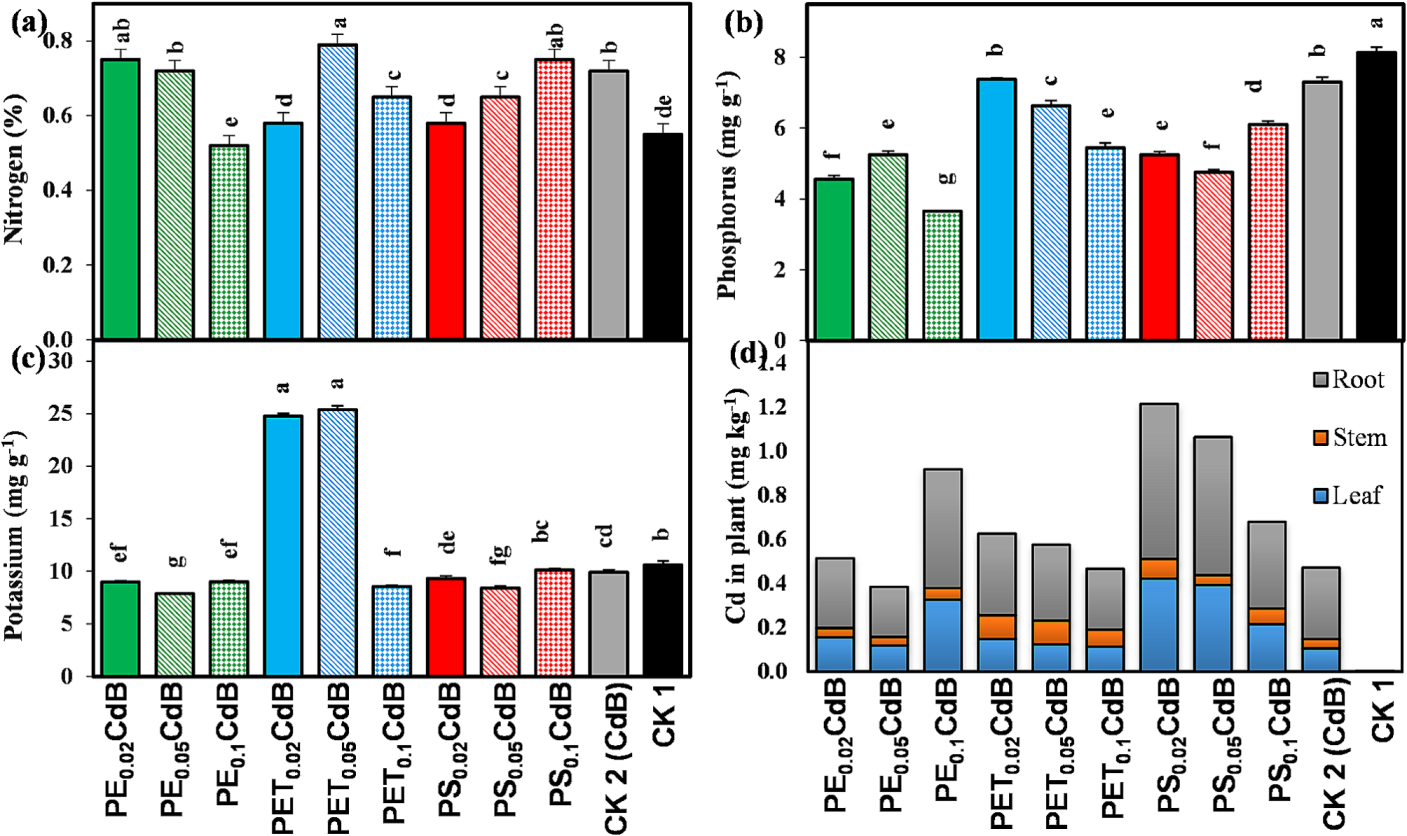
Effects of polyethylene (PE), polyethylene terephthalate (PET), and polystyrene (PS) on (**a**) nitrogen, (**b**) phosphorus, (**c**) potassium, and (**d**) Cadmium contents in the *A. tricolor* shoots in presences of Cd and biochar. Different letters over the bars (a, b, c, d, etc.) indicate significant differences using a one-way ANOVA followed by Duncan’s multiple range test (*p* < 0.05) (*n* = 3)

Compared to CK2, most treatments resulted in a significant reduction in stem nitrogen content (Fig. 3a). Similarly, the stem phosphorus content of *A. tricolor* significantly (*p* < 0.05) decreased across all treatments when compared to CK1 (Fig. 3b). In the case of stem potassium content, all treatments containing MPs showed a marked decrease, except for PET_0.02_CdB and PET_0.05_CdB (Fig. 3c). Furthermore, all three concentrations of PS and the highest concentration of PE (PET_0.1_CdB) led to a significant (*p* < 0.05) increase in cadmium concentrations in plant tissue compared to CK2 (Fig. 3d).

### Bioconcentration and translocation factors

The study shows a significant increase in the BCF of Cd in leaf, stem, and root, and TF-Cd with the additions of different types and concentrations of MPs. The treatment PS_0.02_CdB remarkably increased BCF-leaf by 303%, BCF stem by 114%, BCF root by 117%, and TF by 61%, compared to CK2 (Table 3). It is also observed that all BCF and TF value of Cd are lower than 1 (Table 3).

**Table 3:** Effects of polyethylene (PE), polyethylene terephthalate (PET), and polystyrene (PS) on the bioconcentration factor (BCF) and translocation factor (TF) of *A. Tricolor* in presences of Cd and biochar. Different letters (a, b, c, d, etc.) indicate significant differences using a one-way ANOVA followed by Duncan’s multiple range test (*p* < 0.05) (*n* = 3)

Treatments	BCF-Cd			TF-Cd
	Leaf	Stem	Root	
PE_0.02_CdB	0.052 ± 0.002^e^	0.014 ± 0.0002^fg^	0.106 ± 0.002^g^	0.624 ± 0.007^c^
PE_0.05_CdB	0.04 ± 0.002^f^	0.013 ± 0.0003^g^	0.076 ± 0.002^i^	0.689 ± 0.014^ab^
PE_0.1_CdB	0.109 ± 0.004^c^	0.017 ± 0.0003^e^	0.18 ± 0.002^c^	0.702 ± 0.031^ab^
PET_0.02_CdB	0.049 ± 0.001^e^	0.036 ± 0.0003^a^	0.123 ± 0.003^e^	0.689 ± 0.004^ab^
PET_0.05_CdB	0.041 ± 0.001^f^	0.036 ± 0.0009^a^	0.115 ± 0.001^f^	0.669 ± 0.006^b^
PET_0.1_CdB	0.037 ± 0.001^f^	0.026 ± 0.0006^c^	0.092 ± 0.002^h^	0.689 ± 0.002^ab^
PS_0.02_CdB	0.141 ± 0.003^a^	0.03 ± 0.0006^b^	0.234 ± 0.005^a^	0.728 ± 0.013^a^
PS_0.05_CdB	0.131 ± 0.003^b^	0.015 ± 0.0004^f^	0.208 ± 0.002^b^	0.7 ± 0.02^ab^
PS_0.1_CdB	0.072 ± 0.002^d^	0.023 ± 0.0006^d^	0.131 ± 0.002^d^	0.724 ± 0.02^a^
CK 2 (CdB)	0.035 ± 0.001^f^	0.014 ± 0.0007^g^	0.108 ± 0.001^fg^	0.451 ± 0.001^d^

### Root traits

**Fig. 4:**
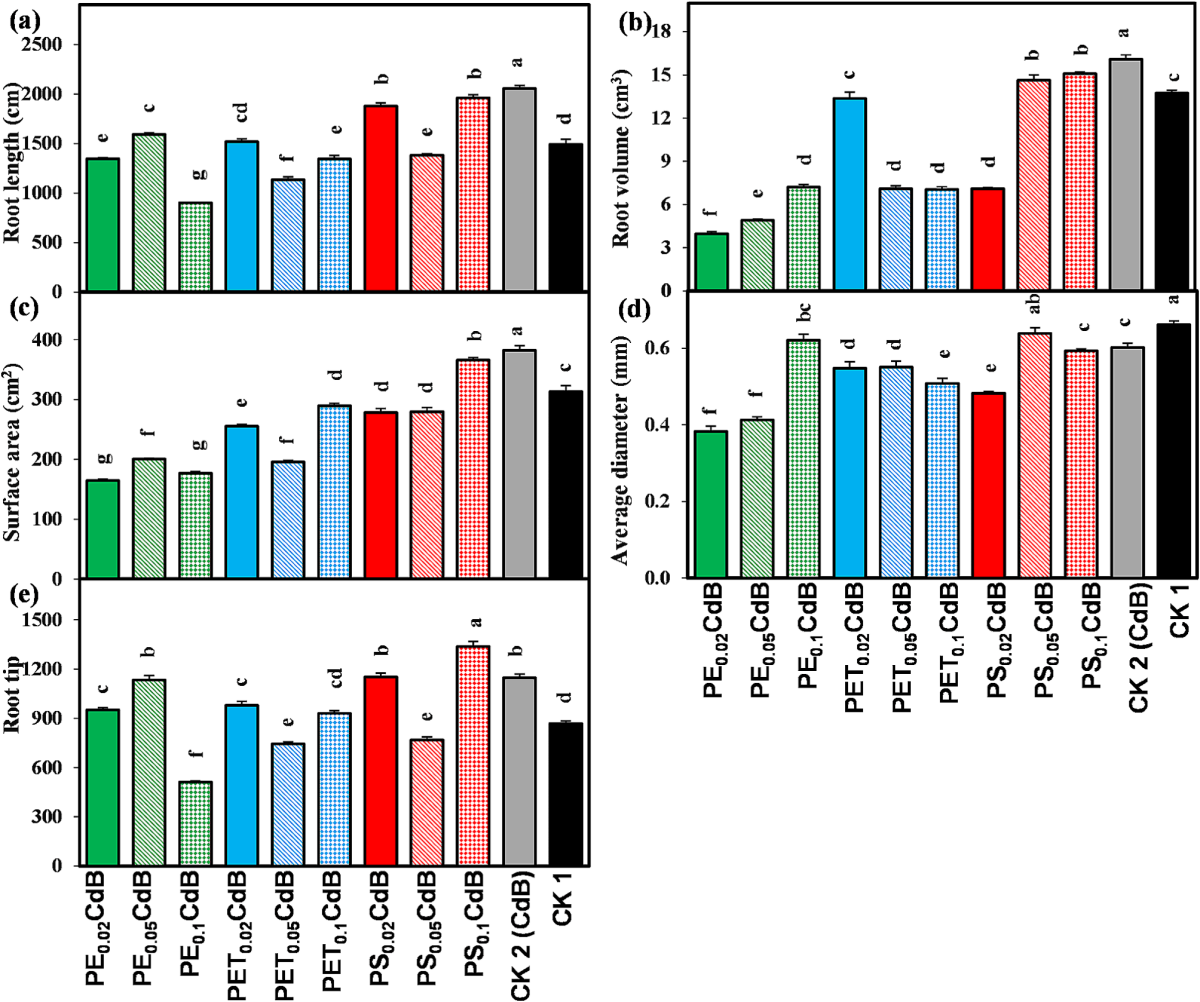
Effects of polyethylene (PE), polyethylene terephthalate (PET), and polystyrene (PS) on (**a**) root length, (**b**) root volume, (**c**) surface area, (**d**) average diameter, and (**e**) root tip number of *A. tricolor* in presences of Cd and biochar (**B**). Different letters over the bars (a, b, c, d, etc.) indicate significant differences using a one-way ANOVA followed by Duncan’s multiple range test (*p* < 0.05) (*n* = 3)

In comparison to CK2, all treatments led to a notable decrease in total root length, root volume, and surface area (Fig. 4a-c). Conversely, we noted the highest average root diameter in CK1 (Fig. 4d). With the exception of treatments PE_0.05_CdB, PS_0.02_CdB, and PS_0.1_CdB, all other treatments significantly reduced the total root tip number of *A. tricolor* compared to CK2 (Fig. 4e).

### Growth assessment of *A. tricolor* in responses to different types and concentrations of MPs in presences of Cd and biochar through Principal component analysis

**Fig. 5:**
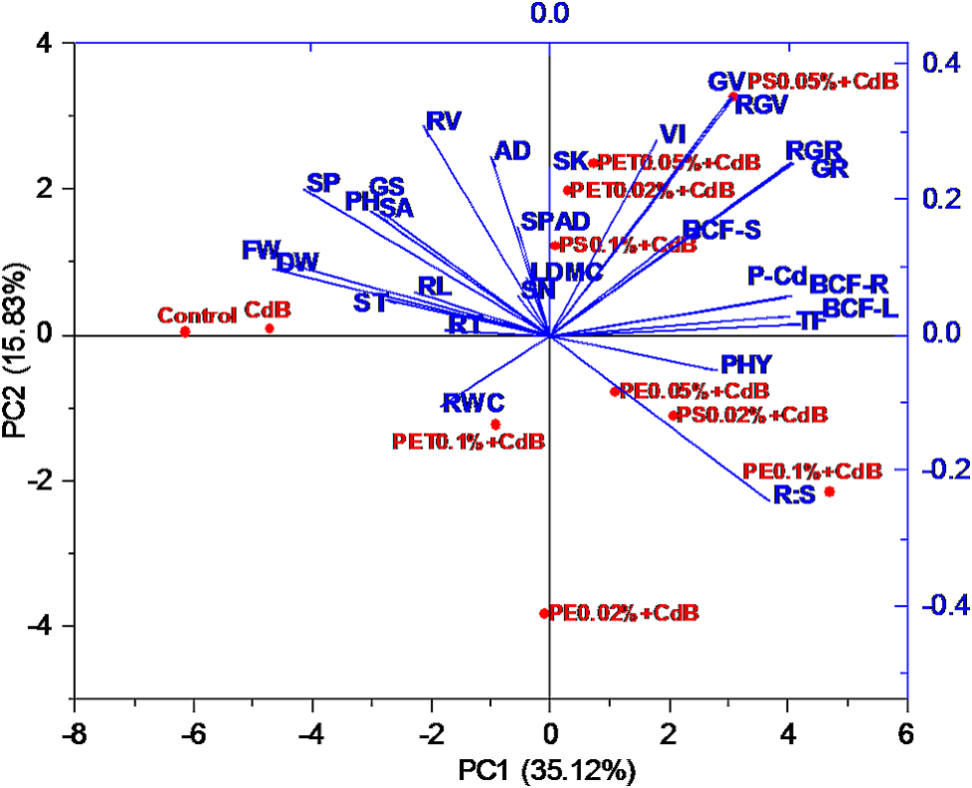
Principal component analysis shows the effect of polyethylene (PE), polyethylene terephthalate (PET), and polystyrene (PS) on the various growth responses of *A. tricolor* in presences of Cd and biochar (**B**)

Principal component analysis (PCA) was conducted to investigate the relationship between different treatments and growth parameters of *A. tricolor* (Fig. 5). It was clearly observed that addition of MPs caused a clear separation of PC1 where treatments without MPs located in the left side of the PCA score plot and positively correlated with plants height, biomass, roots traits and nutrient contents. In contrast, MPs containing treatments located in the right side of the PCA score plot and showed a positive association with germination index, phytotoxicity, bioaccumulation and translocation of Cd (Fig. 5).

### Growth assessment of *A. tricolor* in responses to different types and concentrations of MPs in presences of Cd and biochar through Pearson’s correlation analysis

**Fig. 6:**
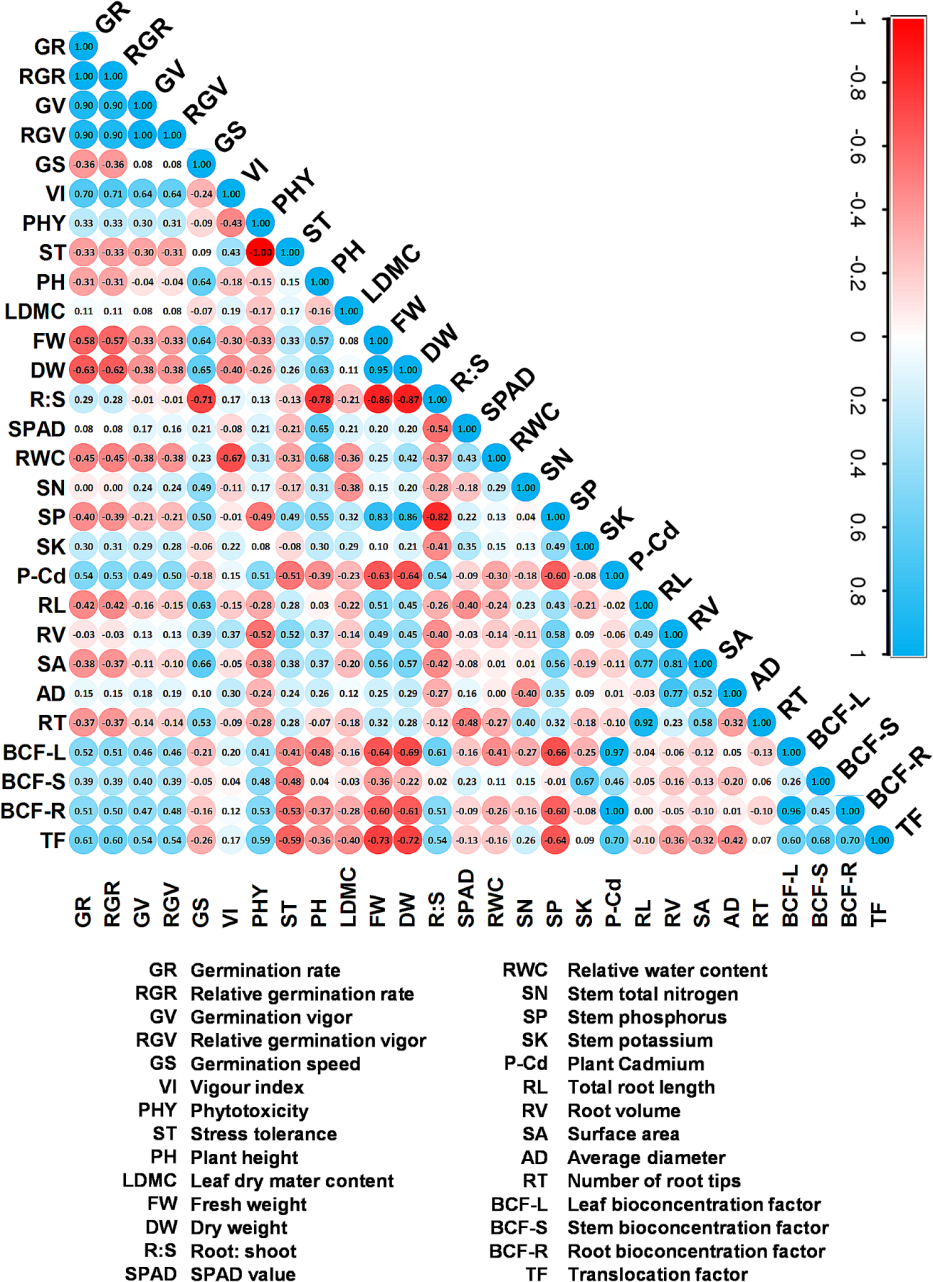
Pearson’s correlation coefficient shows the effect of polyethylene (PE), polyethylene terephthalate (PET), and polystyrene (PS) on the various growth responses of *A. tricolor* in presences of Cd and biochar

Pearson’s correlation analysis was conducted to quantify the relationships among various parameters, encompassing germination, growth attributes, nutrient contents, and heavy metal uptake (Fig. 6).

Germination-related indices (including germination rate, GR; relative germination rate, RGR; germination vigor, GV; relative germination vigor, RGV; and phytotoxicity, PHY) exhibited negative associations with morphological growth attributes (such as plant height, PH; fresh weight, FW; and dry weight, DW), root traits (like root length, RL; root volume, RV; surface area, SA; and root tip number, RT) and positive correlation with BCF and TF-Cd value (Fig. 6). Furthermore, positive associations were observed between SPAD value, relative water content (RWC), and plant nutrient contents such as nitrogen (SN), phosphorus (SP), and potassium (SK) with PH, FW, and DW (Fig. 6). In contrast, heavy metal content (P-Cd) demonstrated negative associations with PH, FW, and DW (Fig. 6).

### Growth assessment of *A. tricolor* in responses to different types and concentrations of MPs in presences of Cd and biochar through heatmap analysis

**Fig. 7:**
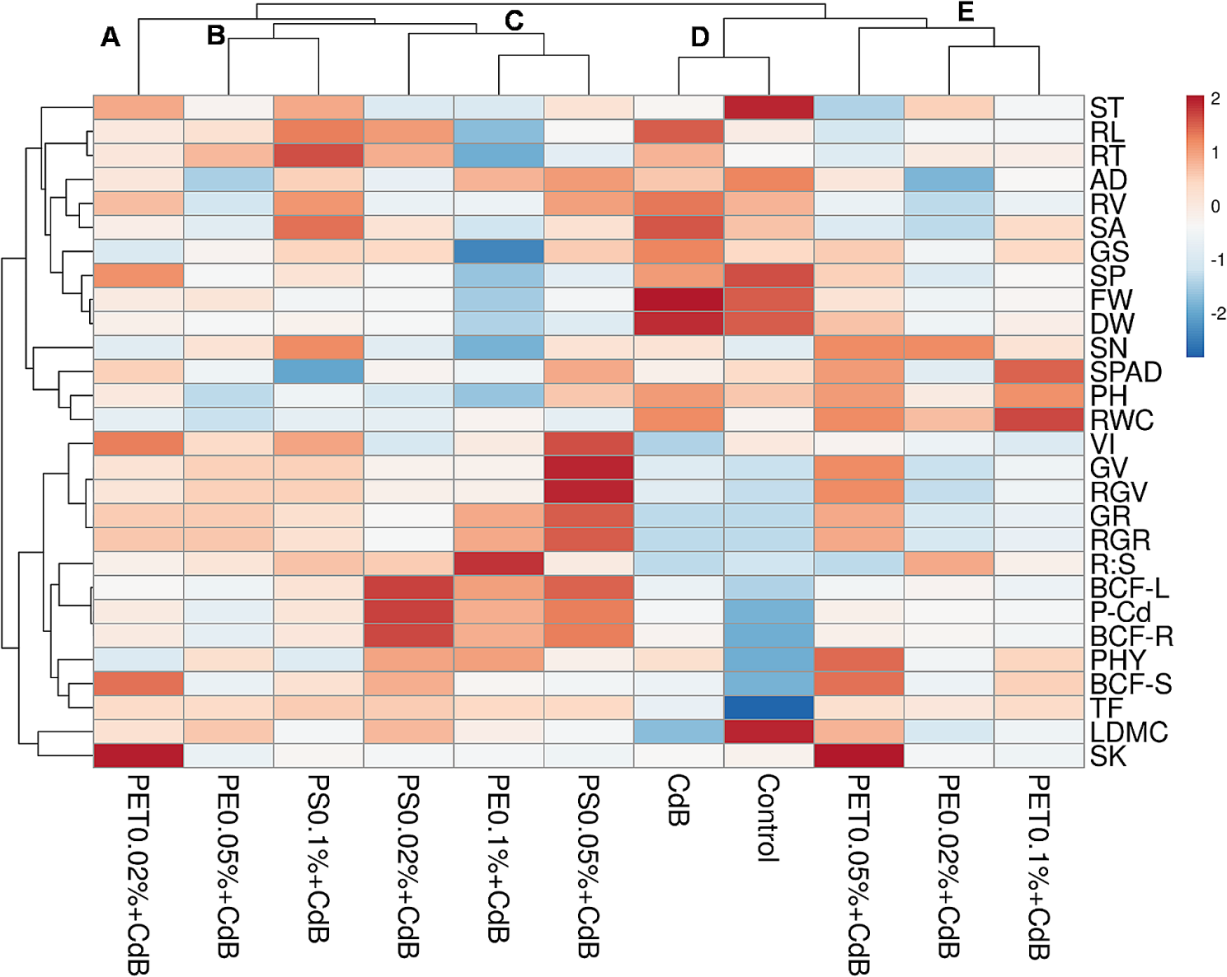
Heatmap shows the effect of polyethylene (PE), polyethylene terephthalate (PET), and polystyrene (PS) on the various growth responses of *A. tricolor* in presences of Cd and biochar (**B**)

In our study heatmap analysis revealed five distinct clusters (Fig. 7). Here, treatment PET_0.02_CdB was clustered in group A and showed higher SK content. Treatments PE_0.05_CdB and PS_0.1_CdB were clustered in group B and displayed better root growth traits. Treatments PS_0.02_CdB, PE_0.1_CdB and PS_0.05_CdB were clustered in group C and produced lower root growth traits but higher germination index, plant Cd accumulation and translocation. Treatments CK1 and CK2 (CdB) were clustered in group D and showed higher values of ST, RL, RT, AD, RV, SA, GS, SP, FW, DW, LDMC and lower levels of germination and Cd accumulation and translocation. Finally, treatments PET_0.05_CdB, PE_0.02_CdB and PET_0.1_CdB were clustered in group E and showed higher levels of SK, SN, SPAD, PH and RWC (Fig. 7).

## Discussion

Microplastics (MPs) have been classified as “emerging” contaminants, and their significant influence on marine ecosystems and the organisms living in them has been extensively studied in the last ten years, highlighting the seriousness of the issue. But there hasn’t been much research on how MPs affect terrestrial plants and agro-crops, especially when it comes to leafy vegetables [9, 46]. Furthermore, research examining the ability of MPs to form bonds with other organic pollutants, including heavy metals and diverse compounds, has been conspicuously limited [9, 13]. To address the gaps in existing research, our study aimed to offers novel insights into how different types and concentrations of MPs in presence of Cd and biochar can affect germination, growth characteristics, nutrient absorption, and heavy metal uptake in terrestrial plants.

Germination is a triphasic process involving initial water uptake (imbibition), stable water absorption with testa ruptures, and subsequent emergence of the radical and hypocotyl [47, 48]. The germination rate serves as a key indicator of a seed’s capacity to germinate after exposure to MPs [40]. While previous studies indicated a negative impact of MP exposure on seed germination percentage [39], our observations revealed a significant enhancement in the germination-related parameters of *A. tricolor* with different types and concentrations of MPs, except for treatments PE_0.02_CdB and PET_0.1_CdB (Fig. 1a-d). This unexpected positive effect may be attributed to the low concentration of MP exposure, aligning with findings by Zhang et al. [49]. In their study, Lian et al. found that polystyrene nanoparticles led to an increase in the activity of α-amylase, which in turn enhanced the germination rate of wheat (*Triticum aestivum* L.) [50]. Moreover, the study conducted by Zhang et al. revealed that rice seed germination rate was enhanced as a result of MP exposure [49]. Germination speed remained unaffected by MP exposure, but lower concentrations of PET and moderate to higher concentrations of PS significantly increased the vigor index (Fig. 1e-f). Conversely, PE did not exhibit a similar trend. Phytotoxicity and stress tolerance of *A. tricolor* increased and decreased, respectively, under MP and Cd exposure, even in the presence of biochar (Fig. 1g-h). Prior research has indicated that different forms of MPs can have varying harmful impacts on plants [35, 51, 52]. These phenomena can be attributed to two factors: (i) the selective affinity of plants towards various types of MPs, and (ii) the variations in toxicity resulting from the diverse degradation capacities and degradation byproducts of MPs. The toxic effects of chemicals on seed germination are intricately linked to their chemical structure, size, shape, and plant species [53]. Therefore, the impact of MPs on seed germination is diverse, necessitating a case-by-case examination.

The reduction in plant height, fresh weight, and dry weight seen in our study (Table 2) can be ascribed to various complex mechanisms associated with the application of MPs. The MPs can physically block plant roots from absorbing nutrients and water, release toxic chemicals during degradation, alter soil microbial communities, hinder root development, cause osmotic stress, disrupt endocrine function, cause nutrient imbalances, and divert energy from growth [54]. Moreover, the existence of MPs in the soil might disturb the production of organic compounds in the plant leaf, including those that contribute to the formation of leaf dry matter. When these factors come together, plants become less healthy overall, with lower biomass and weakened physiological systems. Similarly, Wu et al. found that the use of PS-MPs resulted in a significant reduction in both the biomass and lengths of rice (*Oryza sativa* L.) shoots [55]. Lozano et al. found that the biomass of wild carrot (*Daucus carota* L.) was dramatically boosted by eight distinct forms of MPs [56]. In contrast, Zong et al. found no discernible impact of PS-MPs on the growth of hydroponic wheat (*Triticum aestivum* L.) seedlings [57]. Nevertheless, we saw that administering moderate to high dosages of PET significantly elevated the SPAD value of *A. tricolor*, hence promoting an increase in the height of the examined plant species. The variations in the impact of MPs on plant growth can be ascribed to the specific characteristics such as type, size, and concentration of MPs used [58]. The R/S ratio is regarded as a crucial indication for the allocation of biomass under conditions of environmental stress [59]. The current study observed a considerable rise in the R/S ratio when various types and concentrations of MPs were introduced (Table 2). This finding is consistent with the observation that there is a notable increase in the R/S ratio in wheat upon exposure to polystyrene nanoplastics at concentrations of 0.1%, 1%, and 1 mg L^− 1^ [50].

The uptake of N and K by *A. tricolor* was not affected by the addition of MPs (Fig. 3a and c). In line with our results, Shorobi et al. observed little effects on the absorption of macronutrients (such as N, P, and K) after being exposed to polypropylene MPs [60]. Nevertheless, a noteworthy reduction in P concentration was detected in cherry tomato shoots following exposure to PP-MPs. Similarly, we observed a significant decrease in P content in the shoots of *A. tricolor* when exposed to different types and concentrations of MPs (Fig. 3b). Our study also found that using MPs, namely various concentrations of PET and PS, considerably raised the amount of Cd in *A. tricolor* shoots (Fig. 3d). Specially, PS at 0.02 to 0.05% enhances Cd uptake from the soil and increase heavy metal content in the *A. tricolor*. Which indicate that PS MP might act as vector to carry heavy metal into the plant systems. Similar results were also observed from the PCA, Pearson’s correlation, and heatmap analysis (Figs. 5, 6 and 7). The increase in Cd content with the application of MPs that has been observed could potentially be ascribed to various mechanisms. MPs can cause alterations in the physicochemical qualities of soil, such as pH and organic matter content, which may increase the availability of Cd [61]. Moreover, the creation of complexes between Cd and MPs (Cd-MPs) could enhance the absorption of Cd by plant roots. Additionally, introducing MPs into soil can alter microbial diversity and activity, potentially leading to shifts in key ecosystem processes [62]. Recent studies suggest that MPs may create physical barriers, reducing soil porosity and affecting root-microbe interactions, ultimately disrupting the rhizosphere environment [63]. The degradation of MPs could release harmful chemicals, leading to changes in microbial metabolism or causing a toxic response. Research also indicates that certain types of MPs, such as PET and PS, commonly used in industrial and consumer products, can accumulate in soil and act as vectors for heavy metals and other contaminants [64]. This accumulation might increase Cd bioavailability, affecting microbial health and function. Moreover, the physiological stress response elicited in *A. tricolor* due to the presence of MPs could affect the uptake and movement of essential nutrients, and buildup of Cd. Remarkably, although PET and PS greatly increased the concentration of Cd in the plant system, PE did not have any discernible impact on the uptake of Cd by *A. tricolor*. This implies that different types of MPs may have different effects and that the impact of MPs on the intake of heavy metals cannot be generalized [65].

The BCF and TF values show how well metals are taken up by plants [66]. These values are very important for figuring out how toxic HMs are and how they move from the soil to plants [67]. The experimental data showed that the BCF values for Cd increased when different types and concentrations of MPs were added. This could be attributed to MPs having a high surface area-to-volume ratio, allowing Cd to absorb effectively onto their surfaces. Khalid et al. states that MPs surfaces are vital for adsorbing various heavy metals [68]. The Cd in the water can bind to MPs through physical adsorption. Factors affecting this process include surface properties of the MPs (e.g., surface charge, hydrophobicity), chemical properties of heavy metals, and environmental conditions like pH and temperature. Furthermore, MPs in the rhizosphere have the ability to change the physical and chemical characteristics of the soil environment, impacting the movement and accessibility of Cd ions. They can affect microbial activity and root exudation patterns, thereby influencing the uptake of Cd by plant roots. Some types of MPs can increase the release of Cd from soil particles or organic matter, making it more available for root absorption and transportation to the shoots. Azeem et al. found that PET-MPs can act as a carrier for heavy metals in a simulated plant rhizosphere [69], which aligns with the results of our study (Table 3).

Under the influence of biochar (CK2), there is a notable enhancement in the total length, volume, and surface area of roots (Fig. 4a-c). However, the addition of MP substantially reduces these attributes. The porous structure of biochar enhances water retention and promotes beneficial microbial activity, leading to good effects on plant root parameters [70]. The high Cation Exchange Capacity of biochar enhances nutrient availability, stimulates root exudation, regulates pH levels, enhances soil structure, and facilitates mycorrhizal connections, all of which collectively promote vigorous root development [71]. Nevertheless, the introduction of MPs presents difficulties as their tangible existence might impede the growth of roots, and the chemicals emitted during decomposition may have a detrimental impact on root well-being [72]. Furthermore, MPs possess the capacity to modify the movement of water and the composition of soil microbial communities, potentially resulting in a multifaceted and detrimental impact on plant roots [73]. Notably, the use of MPs, Cd, and biochar all result in a significant reduction in the average diameter of roots (Fig. 4d). Biochar has the potential to enhance soil structure by improving water retention and aeration. However, it may also have the capacity to modify soil structure and impact the patterns of root growth, depending on certain circumstances. For example, if the soil gets too compressed as a result of biochar, it may restrict the growth of roots and lead to a decrease in the average diameter of roots. In line with our results, Liu et al. documented that the addition of biochar significantly boosts the density of root biomass while reducing the average diameter of tobacco roots [74]. In a study conducted by De Souza Machado et al. revealed that the use of different types of MPs had a substantial impact on the growth of spring onions (*Allium fistulosum* L.) roots [51]. Specifically, the total length of the roots increased, but the average diameter of the roots decreased.

Our study reveals significant insights into the effects of MPs on *A. tricolor*, indicating the variability in plant responses to different types and concentrations of MPs. While previous research predominantly suggested a negative impact of MPs on seed germination, our results indicated that certain MPs could enhance germination, with others having no effect or even a negative impact, suggesting a more complex relationship between MPs and plant growth. A novel aspect of our work is the role of MPs as vectors for Cd. We observed that specific types of MPs led to significant Cd accumulation in *A. tricolor* shoots, implying that MPs can facilitate the uptake of heavy metals from soil into plant tissues, raising potential food safety concerns. Additionally, our examination of MPs effects on root structure provided novel insights into how MPs could disrupt plant physiology and soil health, as evidenced by reduced root diameter and changes in root biomass. However, our study has certain limitations, as the findings were obtained from a controlled experiment that involved only a limited range of MP types, Cd concentration, and biochar applications. In real-world settings, field soils typically contain a broader spectrum of MPs and heavy metals, suggesting that an open field study might produce different results due to increased environmental variability. Conditions such as fluctuating temperature, humidity, and soil moisture could impact plant growth and MPs behavior. The presence of diverse soil microbes and natural water movement could influence MPs and heavy metal mobility, affecting their interactions and potential leaching [2]. To address these complexities, future studies should prioritize the collection of soil samples from actual areas contaminated with MPs and heavy metals or conduct long-term field experiments in such contaminated environments. This approach will offer a more realistic depiction of how MPs and heavy metals affect plant growth and overall ecosystem health in real-world scenarios, allowing for a deeper understanding of their combined impact on terrestrial ecosystems.

## Conclusion

This study uncovers important interactions between MPs, biochar, and heavy metal (specifically Cd) and their impact on the growth of *A. tricolor*. Our findings suggest that MPs, depending on their type and concentration, can have varying effects on plant germination and growth, with potential to either unexpectedly promote germination rate or hinder plant growth. Notably, PS MPs can facilitate Cd uptake in plants, raising concerns about food safety. Biochar, a sustainable soil amendment, generally promoted root growth, but its benefits were reduced in the presence of MPs and Cd, indicating a complex interplay between these factors. This study underscores the importance of understanding these interactions for sustainable agriculture. Future research should explore different MPs types and concentrations, optimize biochar use to mitigate pollutants, and explore broader implications for sustainable food production, contributing to more effective and sustainable agricultural practices.

## Data Availability

All data generated or analysed during this study are included in this published article.
